# Collagen depletion, not keloid formation, defines lobomycosis lesions: a paired skin analysis

**DOI:** 10.1016/j.mmcr.2026.100787

**Published:** 2026-04-01

**Authors:** Franciely G. Gonçalves, Juliana M. Veridiano, Nuha A. Dsouki, Maria Aparecida S. Pinhal, Gabriel Z. Laporta

**Affiliations:** a Graduate Program in Health Sciences, FMABC University Center, Santo André, SP, 09060-650, Brazil; b Center for Health Sciences and Sports, Federal University of Acre, Rio Branco, AC, 69920-900, Brazil; c Department of Morphology and Physiology, FMABC University Center, Santo André, SP, 09060-650, Brazil; d Department of Biochemistry, FMABC University Center, Santo André, SP, 09060-650, Brazil; e The USC Infectious Disease Translational Research Center, University of South Carolina, Columbia, SC, 29208, USA

**Keywords:** Lobomycosis, Collagen depletion, Extracellular matrix, Histopathology, Neglected tropical diseases

## Abstract

Lobomycosis is a chronic cutaneous infection caused by the uncultivable fungus *Paracoccidioides lobogeorgii*. Lesions are often described as keloid-like, but the organization of the dermal extracellular matrix remains poorly characterized. We analyzed paired lesional and non-lesional skin from five patients in the Brazilian Amazon using histology and immunohistochemistry. Picrosirius red staining revealed depletion and disorganization of type I collagen in lesional dermis. Immunohistochemistry showed modest differences in biglycan and decorin expression, with spatial redistribution of biglycan near fungal structures. Patients treated with itraconazole showed lesion stabilization. These findings indicate collagen loss rather than true keloid formation.

## 1 Introduction

Lobomycosis (Jorge Lobo's disease) is a chronic granulomatous subcutaneous mycosis endemic to the Amazon Basin, with a high concentration of cases reported in Acre, Brazil [1,2]. The disease is caused by *Paracoccidioides lobogeorgii*, an uncultivable *Paracoccidioides* species, and is acquired through traumatic inoculation in individuals exposed to forest environments [3,4]. Lesions typically evolve slowly over years to decades and predominantly affect rural and indigenous populations [[5], [6], [7]]. Despite longstanding recognition of the disease [[8], [9], [10]], therapeutic responses remain inconsistent and management is challenging [[11], [12], [13]].

Histopathologically, lobomycosis is characterized by dense dermal inflammation containing chains of yeast-like fungal cells [5,7,10,[14], [15], [16]]. Clinically, lesions are frequently described as keloid-like because of their firm, nodular appearance [8,[10], [11], [12],^16^]. However, the structural organization of the affected dermis and the quantitative status of collagen deposition have not been systematically quantified and characterized in terms of extracellular matrix composition [10,12]. Increased collagen accumulation has been assumed based on lesion morphology, but has not been confirmed by extracellular matrix analysis.

Small leucine-rich proteoglycans, particularly decorin and biglycan, regulate collagen fibrillogenesis and tissue mechanical stability [17]. Fungal pathogens can also interact with extracellular matrix components through secreted proteolytic enzymes [18], and exocellular serine-thiol proteinases from *P*. *brasiliensis* illustrate how fungal activity may be modulated by host polysaccharides [19]. Recent clinic-histological studies highlight the importance of tissue organization and fungal burden in lobomycosis lesions [20].

Here, we report a case series of five patients with lobomycosis from the Brazilian Amazon and perform comparative histological and immunohistochemical analyses of collagen, decorin, and biglycan in lesional and non-lesional skin samples. This approach seeks to define the structural and molecular alterations of the dermal matrix in skin affected by *P*. *lobogeorgii* and to determine whether these lesions are characterized by collagen preservation, accumulation, or degradation. This study aims to clarify whether lobomycosis lesions are truly keloid-like or reflect collagen depletion and matrix disorganization.

## 2 Case presentation

Day 0 was defined as the date of histopathological confirmation of lobomycosis by skin biopsy demonstrating characteristic chains of yeast-like fungal cells consistent with *P*. *lobogeorgii* [1,2]. Chronicity was defined as the number of years between Day 0 and the biopsy used for the present histopathological analysis. All five patients were male residents of the western Brazilian Amazon with long-term occupational exposure to forest environments, including farming, rubber tapping, and fishing. Demographic and clinical characteristics are summarized in Table 1.

**Table 1:** Clinical and demographic characteristics of five patients with lobomycosis.

Patient	Age at diagnosis (years)	Age at sample collection (years)	Chronicity (years)^a^	Occupation	Clinical form	Lesion location	Antifungal therapy^b^	Surgery	Follow-up status (2 years post-sample)
1	52	60	08	Farmer	Localized	Left hand	Itraconazole (intermittent)	Yes	Clinical remission with a stable cicatricial lesion
2	62	79	17	Rubber tapper	Disseminated	Right auricle, right leg	Itraconazole (intermittent)	Yes	On itraconazole, stable, pruritus absent
3	65	82	17	Rubber tapper	Localized	Right elbow	Itraconazole (intermittent)	Yes	On itraconazole, small residual nodule
4	60	73	13	Farmer	Disseminated	Right arm, right leg	Itraconazole (intermittent)	Yes	On itraconazole, atrophic lesions, pruritus absent
5	54	72	18	Fisherman	Disseminated	Right thigh, right gluteal sulcus, left arm	Itraconazole (intermittent)	Yes	On itraconazole, residual lesions, follow-up ongoing

a Chronicity refers to the time interval (years) between histopathological confirmation of lobomycosis (Day 0) and the skin biopsy collected for the present histopathological analysis.

b Itraconazole therapy; some patients historically received multibacillary multidrug therapy for leprosy (rifampicin, clofazimine, and dapsone) due to clinical overlap with lobomycosis [13].

At Day 0, all patients presented with slowly enlarging nodular cutaneous lesions with a keloid-like appearance [1,2]. Two patients had localized disease characterized by a solitary lesion, whereas three exhibited disseminated cutaneous involvement affecting multiple anatomical sites, including upper limbs, lower limbs, gluteal region, and auricular pavilion. No systemic manifestations were documented, and pruritus was intermittently reported, particularly during periods of lesion enlargement. Prior to Day 0, patients reported progressive lesion growth for several years (approximately Day −2 to −10) without spontaneous regression.

Therapeutic management following Day 0 varied across patients and over time. Itraconazole therapy was introduced at different time points during long-term follow-up, typically at doses of 200–300 mg/day. Surgical excision of nodular lesions was performed in all patients at various stages of disease evolution, generally after partial lesion atrophy under medical therapy (Table 1). Adherence to antifungal therapy was irregular in several cases, with intermittent interruptions. Periods of treatment interruption were frequently accompanied by lesion progression and recurrence of pruritus, whereas resumption of therapy was associated with partial stabilization in most instances.

Skin samples for histological and immunohistochemical analyses were obtained between Day +8 and Day +18 years by dermatological punch biopsy. Paired specimens included lesional skin from lobomycosis lesions and non-lesional skin from clinically unaffected areas of the same patients, serving as internal controls. All specimens were fixed in 10% neutral-buffered formalin, routinely processed, paraffin-embedded, and sectioned.

**Fig. 1:**
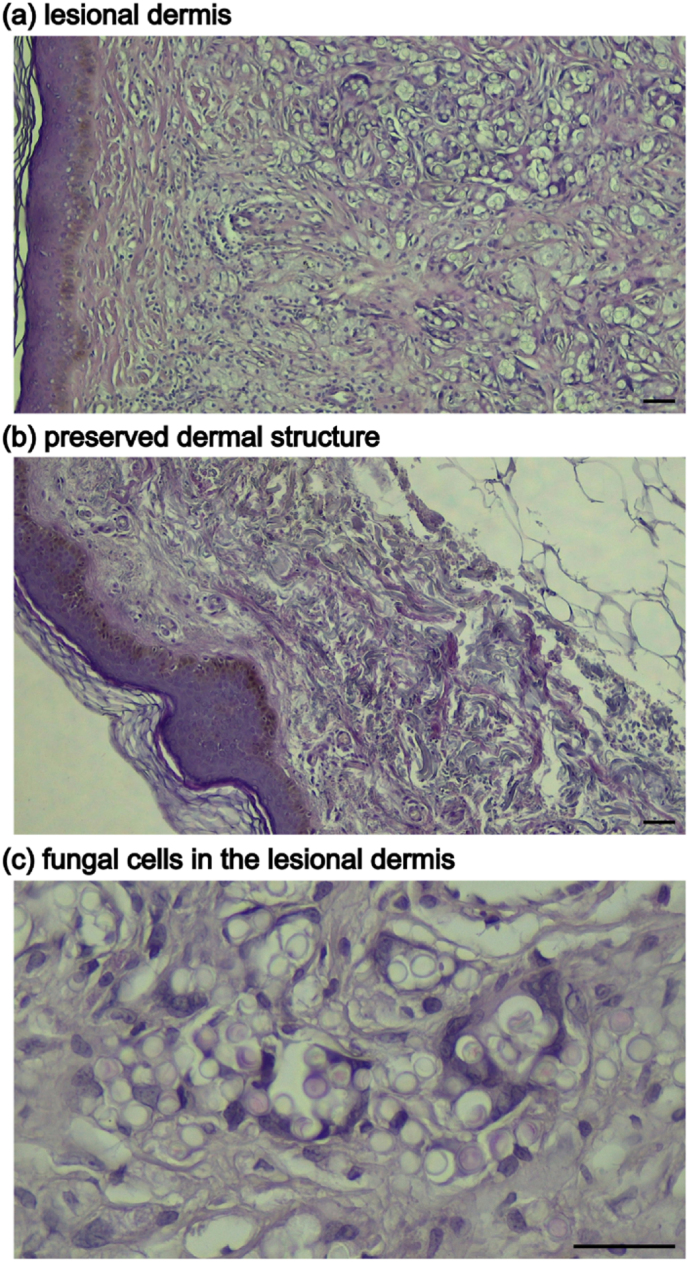
**Histopathological alterations in lobomycosis lesions**. (a) Hematoxylin–eosin staining of lesional dermis showing disrupted dermal architecture with fragmented collagen fibers. (b) Paired non-lesional skin showing preserved dermal structure. (c) Periodic acid–Schiff staining highlighting chains of yeast-like fungal cells within the dermis. Representative micrographs from patient #5 (scale bars = 100 μm).

Hematoxylin–eosin staining revealed marked structural alterations in lesional dermis compared with paired non-lesional skin. Lesional samples showed fragmentation and loss of the normal dense collagen network, indicating pronounced remodeling of the dermal extracellular matrix. In contrast, non-lesional skin preserved the typical collagen architecture of healthy dermis (Fig. 1a and b). Quantitative morphometric analysis further showed that dermal thickness was markedly greater in lesional skin (mean 225 ± 89 μm) compared with paired non-lesional skin (mean 73 ± 69 μm), consistent with extensive structural remodeling of the dermis (Supplementary Fig. 1). Dermal thickness was measured in scanned hematoxylin–eosin-stained sections using an Olympus® slide scanner (Hachioji, Tokyo, Japan) associated with the Learning Machine system. Measurements were defined as the distance from the beginning of the papillary dermis to the end of the reticular dermis and were obtained using the Straight-Line tool in ImageJ.

Periodic acid–Schiff staining demonstrated abundant chains of yeast-like fungal cells within the dermis, frequently located in areas with disrupted extracellular matrix organization (Fig. 1c).

**Fig. 2:**
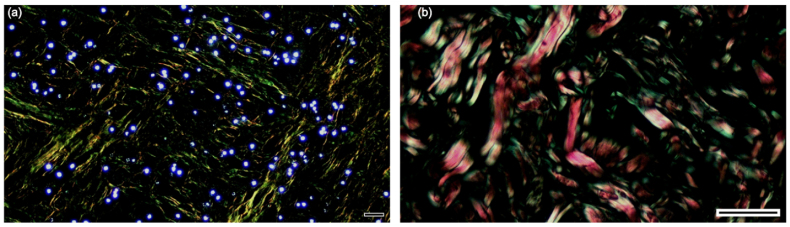
**Collagen fiber organization assessed by Picrosirius red staining under polarized light microscopy**. (a) Lesional dermis showing reduced birefringence intensity, disorganized collagen fibers, and the presence of fungal cells. (b) Paired non-lesional dermis showing a dense and well-organized collagen network. Type I collagen appears as red–orange birefringent fibers, whereas type III collagen appears green. Representative images from patient #5 (scale bars = 100 μm).

Picrosirius red staining under polarized light microscopy revealed substantial alterations in collagen fiber organization. Lesional dermis exhibited reduced birefringence intensity and disorganization of collagen fibers compared with paired control skin. Quantitative analysis showed a marked and consistent reduction in the percentage area of type I collagen in lesional tissue (mean 10% vs. 2), while type III collagen showed a less pronounced decreasing trend (29% vs. 17%) (Fig. 2a and b; Supplementary Table 1). Histological images for collagen analysis were obtained under polarized light microscopy using a Nikon Eclipse E200 (Nikon®, Tokyo, Japan) at 400 × magnification. Quantification of collagen fibers was performed using ImageJ with the Color Threshold tool, allowing differentiation between type I (red–orange) and type III (green) collagen.

**Fig. 3:**
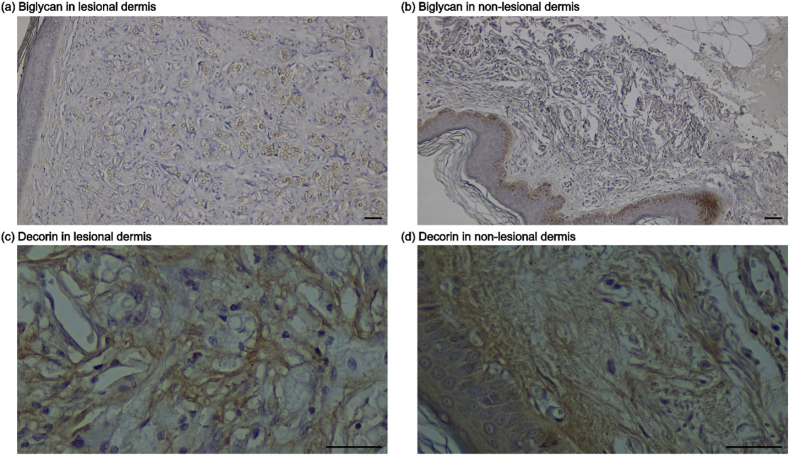
**Immunohistochemical detection of extracellular matrix proteoglycans in lobomycosis lesions**. (a,b) Biglycan staining showing comparable overall expression levels but altered spatial distribution in lesional dermis, with increased localization near fungal structures. (c,d) Decorin staining showing relatively preserved distribution patterns between lesional and non-lesional skin. Representative images from patient #5 (scale bars = 100 μm).

Immunohistochemical analysis did not demonstrate a consistent quantitative reduction of biglycan or decorin in lesional dermis compared with paired non-lesional skin. Quantitative analysis showed only modest differences in the percentage area of both proteoglycans between groups (Supplementary Table 2). However, qualitative assessment revealed a distinct redistribution of biglycan in lesional dermis, with increased localization in areas adjacent to fungal structures (Fig. 3a and b). In contrast, decorin showed no consistent spatial alteration and maintained a diffuse distribution pattern across dermal compartments (Fig. 3c and d).

At the last clinical follow-up (approximately Day +10 to +20 years after diagnosis), one patient was in clinical remission without ongoing antifungal therapy, presenting a stable cicatricial lesion. The remaining four patients continued itraconazole treatment with stable disease, characterized by atrophic or residual lesions, including small nodules. The most consistent clinical improvements were reduction of pruritus and progressive lesion flattening during sustained therapy. Recurrence was mainly observed after treatment interruption.

## 3 Discussion

This study shows that lobomycosis lesions are defined by marked remodeling of the dermal extracellular matrix, with loss and disorganization of collagen rather than the excessive deposition seen in true keloids [8,16]. Comparative analysis of paired lesional and non-lesional skin confirmed disruption of dermal architecture and fragmentation of the collagen network. Picrosirius red staining demonstrated reduced birefringence and decreased type I collagen, supporting substantial alterations in collagen organization. Together, these findings indicate that the keloid-like appearance of lobomycosis lesions reflects collagen depletion and impaired matrix organization rather than true keloid formation.

The observed changes in collagen architecture likely relate to alterations in extracellular matrix proteoglycans, which regulate collagen fibrillogenesis and fiber organization [17,19]. Quantitative analysis showed only modest differences in the overall expression of biglycan and decorin between lesional and non-lesional skin. However, qualitative assessment revealed a distinct spatial redistribution of biglycan in lesional dermis, with increased localization in areas adjacent to fungal structures, suggesting that spatial redistribution, rather than overall depletion, characterizes biglycan alterations in lobomycosis lesions. In contrast, decorin exhibited a more diffuse distribution pattern without consistent spatial changes between lesional and non-lesional tissue. These findings suggest that impaired matrix organization may arise not only from quantitative changes in extracellular matrix components but also from their spatial reorganization within the dermis. In addition, abundant yeast-like fungal cells were frequently observed in areas of disrupted matrix, supporting the presence of a localized microenvironment in which fungal persistence and extracellular matrix remodeling are closely linked [18]. This spatial association raises the possibility that fungal–matrix interactions contribute directly to the reorganization of structural components of the dermis.

Clinically, lobomycosis remains challenging to manage. Surgical excision may reduce lesion burden but recurrence is common, and antifungal therapy such as itraconazole produces variable responses [11]. In this series, patients showed stabilization rather than complete resolution of lesions, and treatment interruptions were associated with lesion progression.

Chronic lesions have occasionally been linked to malignant transformation [15]; although not observed in this cohort, the extensive extracellular matrix remodeling highlights the potential for long-term structural alterations in chronically affected skin.

Limitations of this study include the small number of patients and the cross-sectional design, which precludes temporal assessment of matrix remodeling. Nonetheless, the use of paired lesional and non-lesional samples provides robust internal controls.

In conclusion, lobomycosis lesions are characterized by collagen depletion and disorganization of the dermal extracellular matrix. Rather than representing true keloids, these lesions reflect a state of impaired collagen assembly and matrix remodeling associated with persistent fungal infection. These findings highlight extracellular matrix remodeling as a central feature of lobomycosis pathogenesis and provide a structural framework for future studies investigating host–pathogen interactions in this neglected tropical mycosis.

## CRediT authorship contribution statement

**Franciely G. Gonçalves:** Conceptualization, Data curation, Investigation, Methodology, Visualization, Writing – review & editing. **Juliana M. Veridiano:** Data curation, Investigation, Methodology, Writing – review & editing. **Nuha A. Dsouki:** Data curation, Formal analysis, Investigation, Methodology, Visualization, Writing – review & editing. **Maria Aparecida S. Pinhal:** Methodology, Supervision, Writing – review & editing. **Gabriel Z. Laporta:** Conceptualization, Formal analysis, Funding acquisition, Project administration, Supervision, Writing – original draft.

## Consent

Written informed consent was obtained from the patient or legal guardian(s) for publication of this case report and accompanying images. A copy of the written consent is available for review by the Editor-in-Chief of this journal on request.

## Funding source

This work was partially supported by the USC Infectious Disease Translational Research Center (USC parent project 80005643).

## Declaration of competing interest

The authors declare no competing interests.
