# Supplementary material for: Collagen depletion, not keloid formation, defines lobomycosis lesions: a paired skin analysis
Source: Med Mycol Case Rep. 2026 Apr 1;52:100787. doi: 10.1016/j.mmcr.2026.100787 (PMC13085013; doi:10.1016/j.mmcr.2026.100787)
Supplement: Multimedia component 1 [file mmc1.docx]

**Supplementary Figure 1**

Quantitative comparison of dermal thickness between paired non-lesional and lesional skin samples


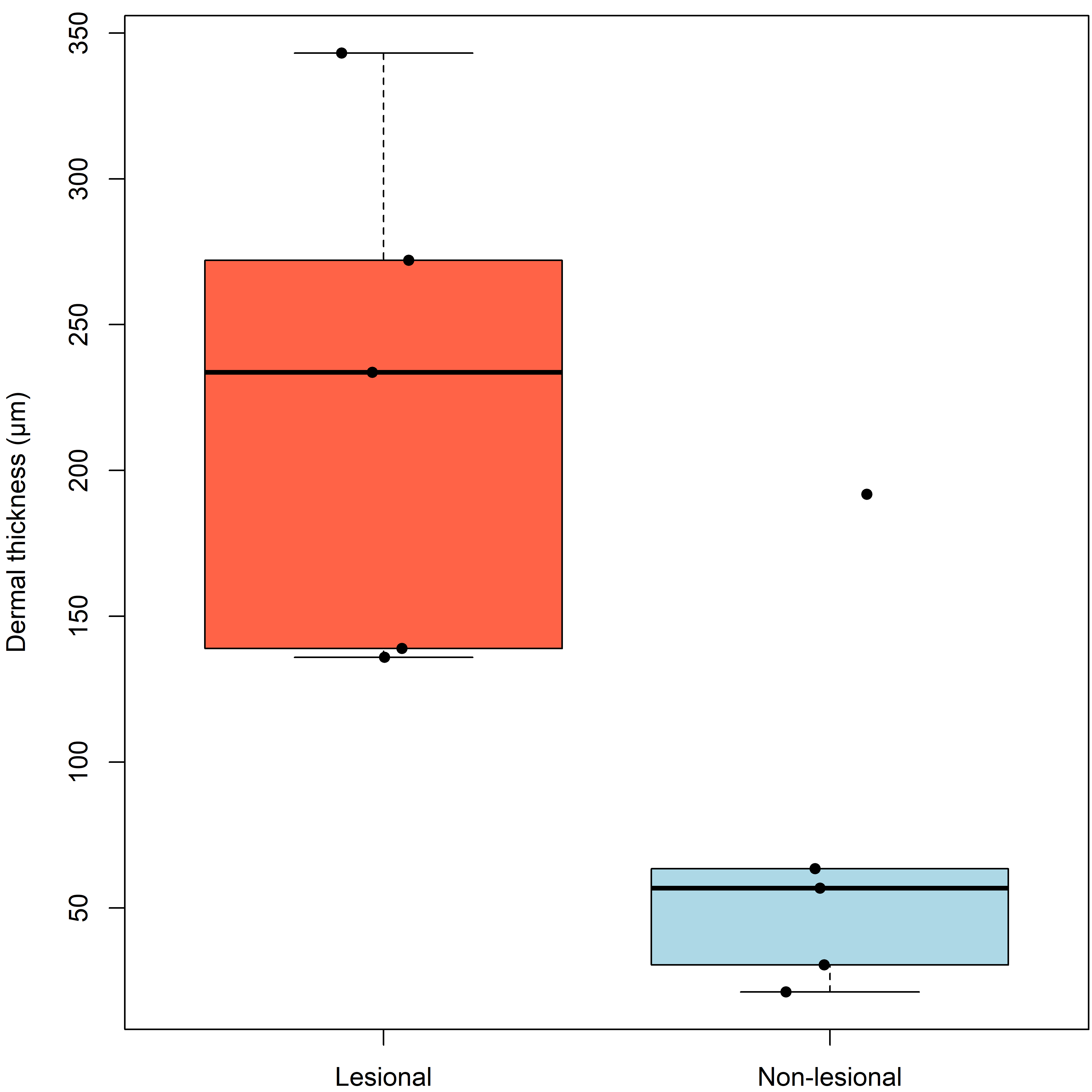


Boxplots show the distribution of dermal thickness (µm), defined as the distance from the beginning of the papillary dermis to the end of the reticular dermis. Values represent the mean of four independent measurements per patient obtained from hematoxylin–eosin-stained sections (n = 5 patients per group). Lesional dermis exhibited markedly greater thickness compared with paired non-lesional skin.

**Supplementary Table 1**

Quantification of collagen fibers in paired skin samples using Picrosirius red staining

| **Patient**^1^ | **Type I Control (%)** | **Type I Lesion (%)** | **Type III Control (%)** | **Type III Lesion (%)** |
| --- | --- | --- | --- | --- |
| 1 | 12.41 | 2.92 | 28.54 | 31.47 |
| 2 | 3.96 | 0.94 | 14.29 | 12.23 |
| 3 | 5.92 | 3.75 | 32.17 | 11.58 |
| 4 | 11.89 | 1.63 | 48.91 | 11.34 |
| 5 | 16.82 | 2.08 | 20.90 | 20.03 |

^1^Values represent the percentage area (%) of red–orange (type I collagen) and green (type III collagen) birefringence, quantified from five randomly selected fields per patient using ImageJ color threshold analysis

**Supplementary Table 2**

Quantitative analysis of extracellular matrix proteoglycans in paired skin samples

| **Patient**^1^ | **Biglycan Control (%)** | **Biglycan Lesion (%)** | **Decorin Control (%)** | **Decorin Lesion (%)** |
| --- | --- | --- | --- | --- |
| 1 | 33.05 | 39.99 | 44.60 | 40.15 |
| 2 | 28.89 | 29.48 | 38.48 | 27.63 |
| 3 | 33.78 | 29.95 | 37.69 | 40.62 |
| 4 | 38.98 | 33.14 | 49.54 | 25.92 |
| 5 | 35.55 | 26.49 | 40.89 | 42.86 |

^1^Values represent the percentage area (%) of immunohistochemical staining for biglycan and decorin, quantified from four randomly selected fields per patient using ImageJ
